# Shock index increase from the field to the emergency room is associated with higher odds of massive transfusion in trauma patients with stable blood pressure: A cross-sectional analysis

**DOI:** 10.1371/journal.pone.0216153

**Published:** 2019-04-25

**Authors:** Shao-Chun Wu, Cheng-Shyuan Rau, Spencer C. H. Kuo, Shiun-Yuan Hsu, Hsiao-Yun Hsieh, Ching-Hua Hsieh

**Affiliations:** 1 Department of Anesthesiology, Kaohsiung Chang Gung Memorial Hospital, Chang Gung University and College of Medicine, Kaohsiung, Taiwan; 2 Department of Neurosurgery, Kaohsiung Chang Gung Memorial Hospital, Chang Gung University and College of Medicine, Kaohsiung, Taiwan; 3 Department of Plastic Surgery, Kaohsiung Chang Gung Memorial Hospital, Chang Gung University and College of Medicine, Kaohsiung, Taiwan; 4 Department of Trauma Surgery, Kaohsiung Chang Gung Memorial Hospital, Chang Gung University and College of Medicine, Kaohsiung, Taiwan; University of Mississippi Medical Center, UNITED STATES

## Abstract

**Background:**

The shock index (SI) is defined as the ratio of heart rate/systolic blood pressure. This study aimed to determine the performance of delta shock index (ΔSI), a difference between SI upon arrival at the emergency room (ER) and that in the field, in predicting the need for massive transfusion (MT) among adult trauma patients with stable blood pressure.

**Methods:**

This study included registered data from all trauma patients aged 20 years and above who were hospitalized from January 1, 2009 to December 31, 2016. Only patients who were transferred by emergency medical service from the accident site with a systolic blood pressure ≥ 90 mm Hg at the ER were included. The 7,957 enrolled trauma patients were divided into 2 groups, those who had received blood transfusion ≥ 10 U (MT, n = 82) and those who had not (non-MT, n = 7,875). The odds ratios with 95% confidence intervals for the need for MT by a given ΔSI were measured. The plot of specific receiver operating characteristic (ROC) curves was used to evaluate the best cutoff point of ΔSI that could predict the patient’s probability of receiving MT.

**Results:**

ROC curve analysis showed that a ΔSI of 0.06 as the cutoff point had the highest AUC of 0.61, with a sensitivity of 0.415 and specificity of 0.841. Patients with a ΔSI ≥ 0.00 had a significant 1.8-fold increase in need for MT than those patients with a ΔSI less than 0.00 (1.4% vs. 0.8%, p = 0.01). The larger the ΔSI, the higher the odds of need for an MT. Using the cutoff point of ΔSI of 0.06, patients with a ΔSI ≥ 0.06 had a significant 3.7-fold increase in need for MT than those patients with a ΔSI less than 0.06 (2.7% vs. 0.7%, p < 0.001).

**Conclusions:**

This study indicated that, in trauma patients with stable blood pressure at the ER, the accuracy of prediction of the requirement for MT by ΔSI is low. However, the size of the delta is significantly associated with need for MT and a lack of improvement in the patient’s SI at the ER compared to that in the field significantly increases the odds of a need for MT.

## Background

Massive transfusion (MT) in trauma is defined as the transfusion of ten or more units of whole blood or packed red blood cells within the first twenty-four hours of arrival to the hospital [1]. Early recognition of the need for MT is important but still presents a challenge in trauma patients. Vital signs alone have proven insufficient in reliably predicting need for MT [2, 3]. The shock index (SI), the ratio of heart rate (HR)/ systolic blood pressure (SBP), has been considered as a marker for significant injury in trauma patients with hypovolemic shock [4], requirement for transfusion [5], and need for MT [3, 6, 7]. The dynamic change in the ratio of HR to SBP reflect the physiological response of an individual to the trauma injury [8–12]. Previous work has shown that SI is moderately accurate in predicting the need for MT. An SI **≥** 0.95 predict the need for MT with an AUC of 0.760 (sensitivity, 0.563, and specificity, 0.876) [13].

The delta shock index (ΔSI) is the difference between SI at the emergency room (ER) and that in the field. It had been proposed that ΔSI may better account for variation unique to an individual reflecting the hemodynamic change upon a stress such as traumatic injury [14]. SI and ΔSI appear to be superior to HR and SBP in predicting postpartum hemorrhage and need for intervention in peripartum women [14]. The ΔSI was superior to traditional SI to predict the prognosis of the trauma patients [15]. In a study on 2,591 patients, patients with ΔSI > 0.1 required three times higher volumes of blood transfusion than those who had a ΔSI ≤ 0.1 [15]. Given that the identification of patients with need for MT is crucial as prompt treatment improves the prognosis [7], the need for MT might be missed in trauma patients with stable blood pressure at the ER. Therefore, the objective of this study was to evaluate the performance of ΔSI in predicting the need for MT in trauma patients with stable blood pressure at the ER.

## Methods

### Ethics statement

The institutional review board of Kaohsiung Chang Gung Memorial Hospital had approved this study (reference number: 201800748B0) and waived the requirement for informed consent.

### Study population

**Fig 1:**
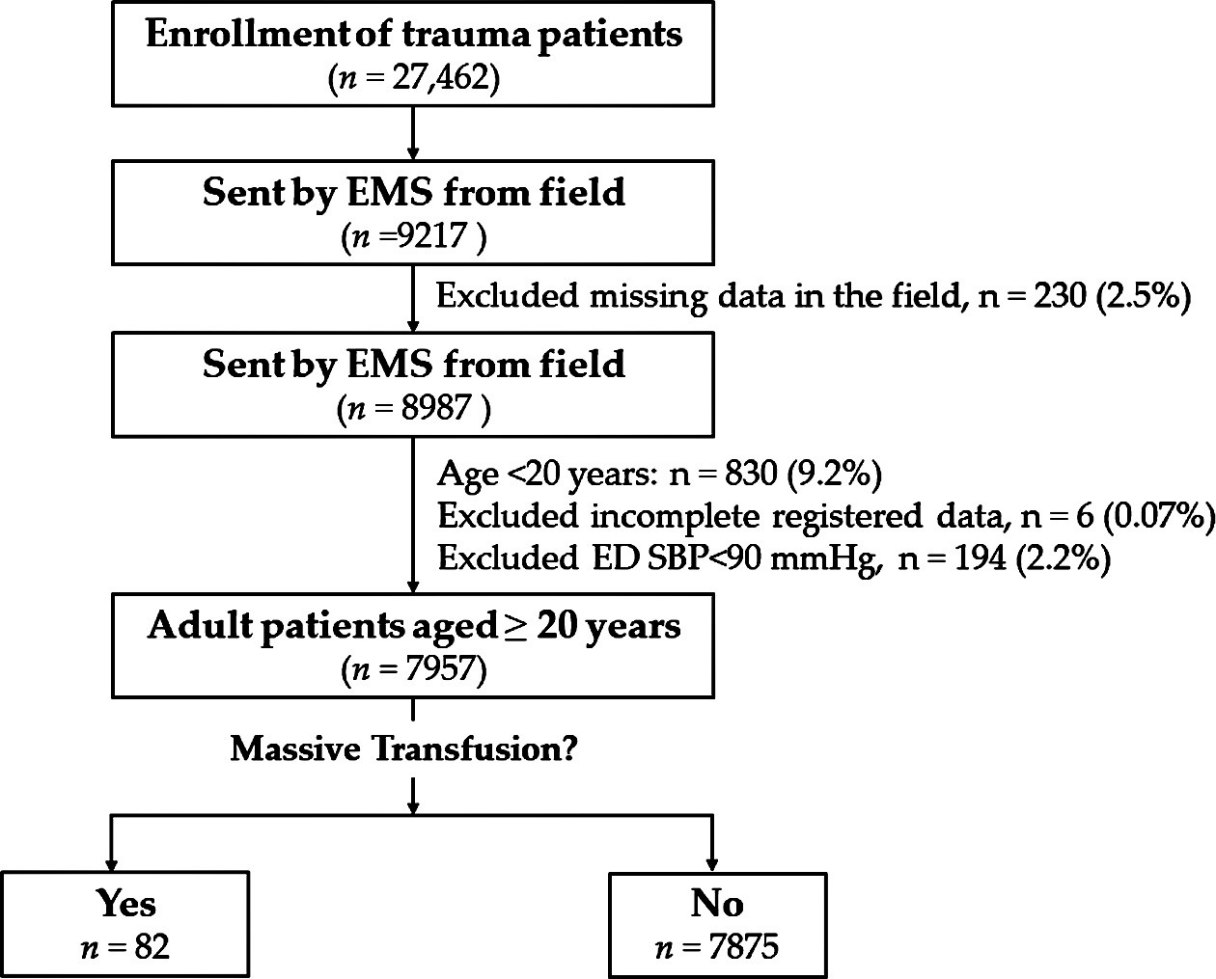
Flowchart of allocation of adult trauma patients into those who had received MT and those who did not.

Patients were identified from the trauma database at Kaohsiung Chang Gung Memorial Hospital, a regional level 1 trauma center in southern Taiwan. Trauma patients were those patients sustained an injury by all trauma causes including motor vehicle accidents, motorcycle accidents, bicycle accidents, pedestrian involved in a traffic accident, fall, and strike by/against objects. To be included in the study, patients had to be injured between January 1, 2009 and December 31, 2016 and fit the inclusion criteria (admitted to the hospital following trauma, age **≥** 20 years, transferred from scene by emergency medical service (EMS), and stable blood pressure (defined by SBP ≥ 90 mmHg) upon arrival. The exclusion criteria included those patients who were transferred from other hospitals or arrived by private vehicles, patients who had missing HR or SBP data in the field, patients aged less than 20 years, patients who had incomplete registered data, and those who had an unstable blood pressure (SBP < 90 mmHg) upon arrival at the ER (Fig 1). A patient receiving transfusion of 10 U or more of whole blood or packed red blood cells within 24 h of arrival at the ER was defined as receiving MT [1]. The vital signs were recorded by the EMS at the field and by the triage nurse upon patients’ arrival to the ER. The Field SI and ER SI were calculated as the ratio of HR to SBP in the field recorded by the EMS and that at the ER recorded by the triage nurse, respectively. The ΔSI was calculated as ER SI minus field SI (ΔSI = ER SI—field SI). In total, 7,957 trauma patients were enrolled in this study and divided into 2 groups, including those who had received blood transfusion ≥ 10 U (MT, n = 82) and those who did not (non-MT, n = 7,875). We retrieved the detailed patient information including age; sex; vital signs; comorbidities, including diabetes mellitus, hypertension (HTN), coronary artery disease, congestive heart failure, and cerebral vascular accident, and end-stage renal disease; injury severity score (ISS), which was expressed as median and interquartile range (IQR, Q1–Q3); units of blood (whole blood and packed red blood cells) transfused within 24 h; and in-hospital mortality.

### Statistical analysis

Statistical analysis was performed using the SPSS software (version 22.0, IBM Corp., Armonk, NY, USA). The requirement of an MT in patients was the primary outcome. The unpaired Student t-test and Mann-Whitney U test were used to analyze normally and non-normally distributed continuous data, which were expressed as mean ± standard deviation and median ± interquartile range (IQR), respectively. Multivariate logistic regression analysis was applied to identify independent effects of univariate predictive variables on the occurrence of MT for trauma adult patients. The odds ratios (ORs) with 95% confidence intervals (CIs) of the associated conditions of the patients and the odds of a need for MT based on a given value of ΔSI were obtained. The plot of specific receiver operating characteristic (ROC) curves was used to evaluate the best cutoff point that could predict the patient’s probability of receiving MT. The cutoff point was derived from ROC curves based on the maximal Youden index, which was calculated as sensitivity + specificity − 1, to reflect the maximal correct classification accuracy. The accuracy of ΔSI in predicting the MT was then calculated in terms of sensitivity and specificity for this cutoff point. An area under the curve (AUC) of more than 0.9 was defined as high accuracy, between 0.9 and 0.7 as moderate accuracy, and less than 0.7 as low accuracy [16]. A P-value < 0.05 indicated statistical significance.

## Results

### Patient characteristics

There was no significant difference between the MT and non-MT groups in terms of age, prevalence rates of comorbidities (except the rates of HTN), field SBP, and field SI (Table 1). A significantly predominant male population and lower ER SBP was found in the MT group than the non-MT group. The MT group had a significantly, higher field HR, ER HR, ER SI, ΔSI, ISS and rate of in-hospital mortality than the non-MT group. The univariate logistic regression analysis (Table 2) demonstrated that a male gender, pre-existing HTN, scene HR, SBP and HR at ER, ER SI, ΔSI, and ISS were significant risk factors for MT in the trauma adult patients. In contrast, age and other pre-existing co-morbidities were not significant risk factors for MT. The multivariate logistic regression analysis revealed that the only ISS (OR, 1.1; 95% CI, 1.11–1.14; p < 0.001) were a significant independent risk factor for MT in trauma adult patients. The SBP, HR, and SI at either the scene or ER as well as the ΔSI were not significant independent risk factors for the requirement of MT.

**Table 1:** Characteristics and outcomes of patients who received and did not received massive blood transfusion. The P-values were calculated using chi-square test for categorical variables as well as unpaired Student t-test and Mann-Whitney U test for normally and non-normally distributed continuous data, respectively.

Variables	MT (Yes) n = 82		MT (No) n = 7,875		P-value
Age (years)	53.9	±19.5	52.7	±19.1	0.561
Gender, n (%)					0.005
Male	57	(69.5)	4224	(53.6)	
Female	25	(30.5)	3651	(46.4)	
Co-morbidities, n (%)					
DM	11	(13.4)	1170	(14.9)	0.759
HTN	14	(17.1)	2179	(27.7)	0.034
CAD	6	(7.3)	262	(3.3)	0.058
CHF	0	(0.0)	54	(0.7)	0.677
CVA	2	(2.4)	280	(3.6)	0.771
ESRD	1	(1.2)	119	(1.5)	>0.99
Field SBP (mmHg)	144.4	±31.1	145.5	±30.5	0.739
ER SBP (mmHg)	142.1	±31.0	153.3	±32.5	0.002
Field HR (beats/min)	93.3	±21.5	57.9	±16.3	0.003
ER HR (beats/min)	98.6	±27.5	85.7	±16.5	<0.001
Field SI	0.7	±0.2	0.6	±0.3	0.271
ER SI	0.7	±0.3	0.6	±0.2	<0.001
ΔSI	0.1	±0.3	-0.1	±0.3	<0.001
ISS (median, IQR)	27	(21–34)	9	(4–10)	<0.001
Mortality, n (%)	39	(47.6)	141	(1.8)	<0.001

CAD = coronary artery disease; CHF = congestive heart failure; CI = confidence interval; CVA = cerebral vascular accident; DM = diabetes mellitus; EMS = Emergent Medical Service; ER = Emergency room; ESRD = end-stage renal disease; HTN = hypertension; HR = Heart Rate; IQR = interquartile range; ISS = injury severity score; MT = massive transfusion; SBP = Systolic Blood Pressure; SI = shock index; ΔSI = delta shock index

**Table 2:** Risk factors influencing massive transfusion in the trauma patients analyzed using univariate and multivariate logistic regression.

	Univariate analysis			Multivariate analysis		
	OR	CI	*P*	OR	CI	*P*
Age (years)	1.0	(0.99–1.02)	*0*.*551*	–		–
Gender (Male)	2.0	(1.23–3.16)	*0*.*005*	1.4	(0.81–2.32)	*0*.*237*
Co-morbidities						
CVA	0.7	(1.17–2.77)	*0*.*589*	–		–
HTN	0.5	(0.30–0.96)	*0*.*035*	0.9	(0.45–1.61)	*0*.*619*
CAD	2.3	(0.99–5.32)	*0*.*053*	–		–
CHF	–		–	–		–
DM	0.9	(0.47–1.68)	*0*.*715*	–		–
ESRD	0.8	(0.11–5.83)	*0*.*830*	–		–
Scene SBP (mmHg)	1.0	(0.99–1.01)	*0*.*739*	–		–
Scene HR (beats/min)	1.0	(1.01–1.03)	*0*.*003*	1.0	(0.99–1.02)	*0*.*689*
ER SBP (mmHg)	1.0	(0.98–1.00)	*0*.*002*	1.0	(0.98–1.02)	*0*.*885*
ER HR (beats/min)	1.0	(1.03–1.05)	*<0*.*001*	1.0	(0.96–1.04)	*0*.*979*
Scene SI	1.3	(0.81–2.08)	*0*.*280*	–		–
ER SI	41.9	(15.22–108.15)	*<0*.*001*	5.1	(0.04–695.28)	*0*.*514*
ΔSI	56.8	(17.12–188.72)	*<0*.*001*	4.7	(0.64–35.05)	*0*.*129*
ISS	1.1	(1.11–1.16)	*<0*.*001*	1.1	(1.10–1.14)	*<0*.*001*

CAD = coronary artery disease; CHF = congestive heart failure; CI = confidence interval; CVA = cerebral vascular accident; DM = diabetes mellitus; ER = emergency room; HTN = hypertension; HR = Heart Rate; IQR = interquartile range; ISS = injury severity score; SBP = Systolic Blood Pressure; SI = shock index; ΔSI = delta shock index

### ΔSI and the need for MT

**Fig 2:**
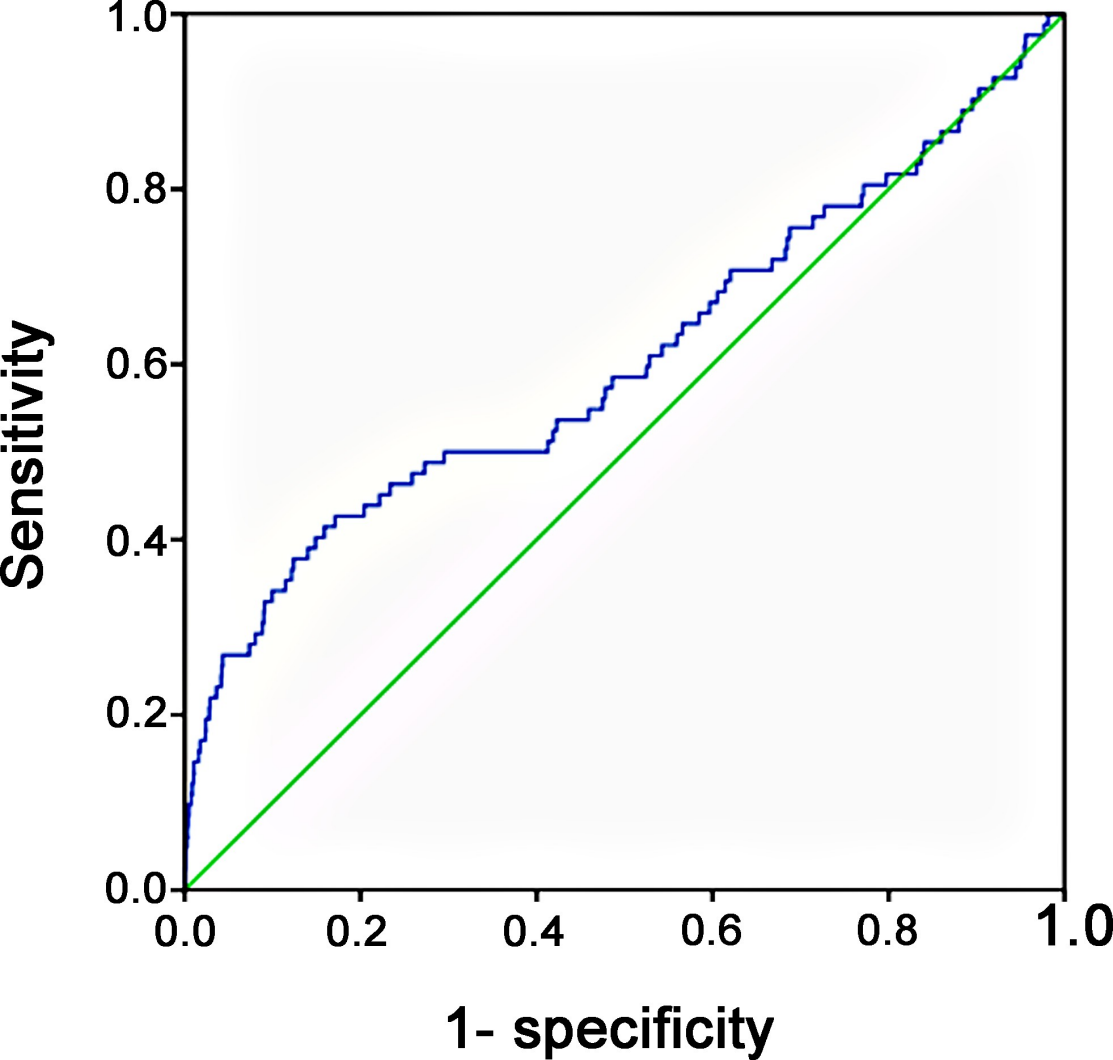
Receiver operating characteristic (ROC) curve analysis to identify the cutoff levels of ΔSI for the requirement of MT.

**Fig 3:**
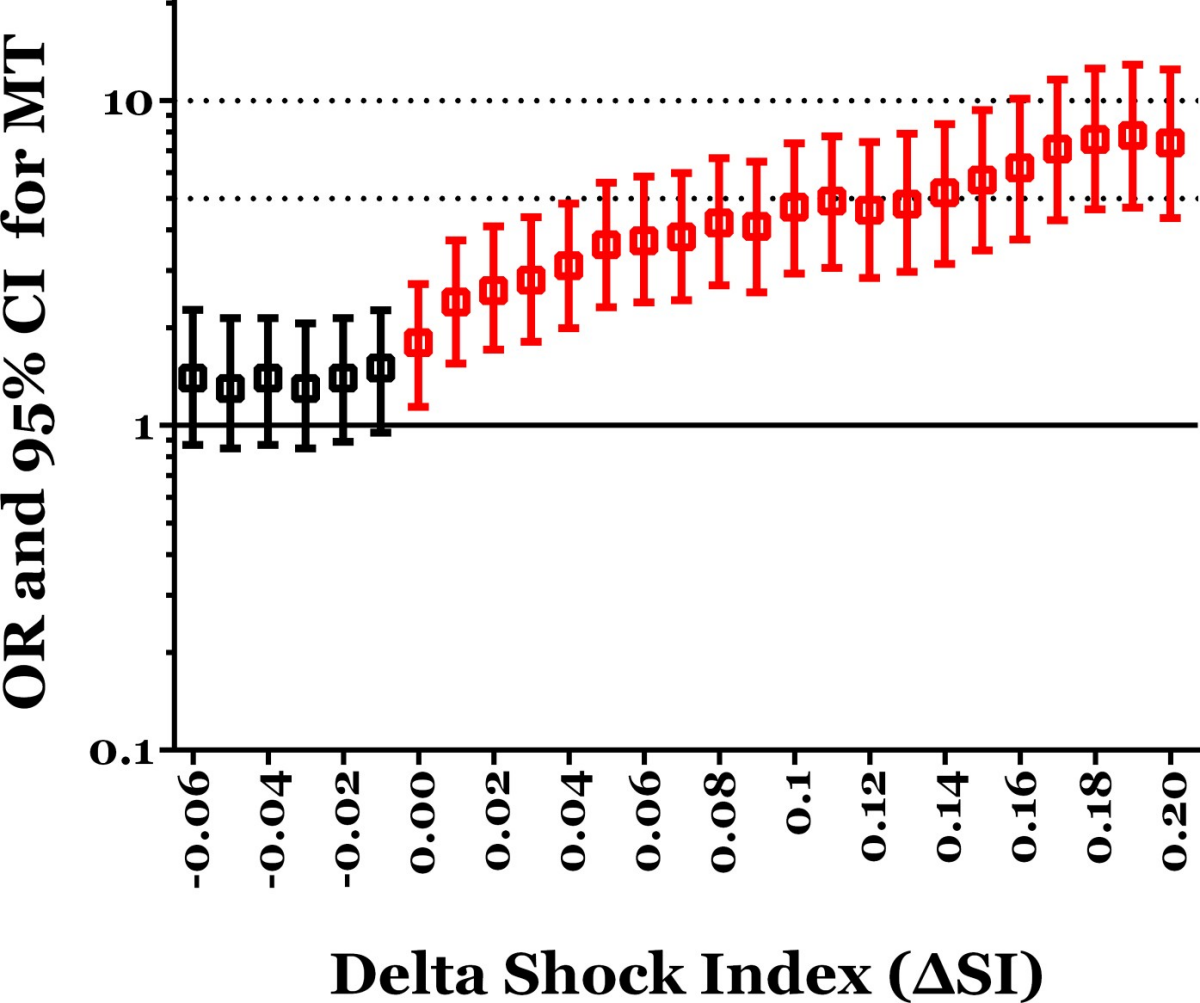
The odds of need for MT in patients with ΔSI ≥ a given value than those with ΔSI < the given value (red color bars indicated a significant difference in the comparison).

According to the ROC curve analysis, a ΔSI of 0.06 as the cutoff point had the highest AUC of 0.61, with a sensitivity of 0.415 and specificity of 0.841 (Fig 2). There was a low accuracy of the discriminating power of ΔSI in predicting the requirement of MT. There was no significant difference in odds of need for MT between patients with a ΔSI ≥ -0.01 and those with a ΔSI < 0.01 (Table 3). Patients with a ΔSI ≥ 0.00 had a significant 1.8-fold need for MT than those with a ΔSI < 0.00 (1.4% vs. 0.8%, p = 0.01). As a given value of ΔSI was higher for separating these trauma patients, the odds of a need for MT in patients with ΔSI ≥ the given value were increased and significantly higher than those with ΔSI less than the given value (Fig 3). At the cutoff point of ΔSI being 0.06, patients with a ΔSI ≥ 0.06 had a significant 3.7-fold need for MT than those with a ΔSI less than 0.06 (2.7% vs. 0.7%, p < 0.001). Furthermore, patients with a ΔSI ≥ 0.20 had a significant 7.4-fold need for MT than those with a ΔSI < 0.20 (5.9% vs. 0.8%, p < 0.001).

**Table 3:** Odds and risk for MT in patients with ΔSI ≥ the defined value than those with ΔSI < the defined value.

Defined value of ΔSI	Patient with ΔSI ≥ defined value			Patient with ΔSI < defined value			OR(95% CI)	P-value
	Patient number (MT)	Patient number (total)	MT % (%)	Patient number (MT)	Patient number (total)	MT % (%)		
-0.06	58	5,033	1.2%	24	2,924	0.8%	1.4(0.87–2.27)	0.158
-0.05	55	4,799	1.2%	27	3,158	0.9%	1.3(0.85–2.14)	0.208
-0.04	52	4,458	1.2%	30	3,499	0.9%	1.4(0.87–2.14)	0.175
-0.03	48	4,107	1.2%	34	3,850	0.9%	1.3(0.85–2.06)	0.207
-0.02	45	3,734	1.2%	37	4,223	0.9%	1.4(0.89–2.14)	0.147
-0.01	42	3,335	1.3%	40	4,622	0.9%	1.5(0.95–2.26)	0.086
0.00	41	2,893	1.4%	41	5,064	0.8%	1.8(1.14–2.72)	0.010
0.01	40	2,277	1.8%	42	5,680	0.7%	2.4(1.55–3.71)	<0.001
0.02	38	1,976	1.9%	44	5,981	0.7%	2.6(1.71–4.10)	<0.001
0.03	36	1,749	2.1%	46	6,208	0.7%	2.8(1.81–4.37)	<0.001
0.04	35	1,563	2.2%	47	6,394	0.7%	3.1(1.99–4.81)	<0.001
0.05	35	1,386	2.5%	47	6,571	0.7%	3.6(2.31–5.59)	<0.001
0.06	33	1,236	2.7%	49	6,721	0.7%	3.7(2.39–5.83)	<0.001
0.07	31	1,113	2.8%	51	6,844	0.7%	3.8(2.43–5.99)	<0.001
0.08	31	1,020	3.0%	51	6,973	0.7%	4.2(2.70–6.65)	<0.001
0.09	28	915	3.1%	54	7,042	0.8%	4.1(2.57–6.48)	<0.001
0.10	28	817	3.4%	54	7,140	0.8%	4.7 (2.93–7.39)	<0.001
0.11	27	749	3.6%	55	7,208	0.8%	4.9(3.05–7.76)	<0.001
0.12	24	673	3.6%	58	7,284	0.8%	4.6(2.84–7.46)	<0.001
0.13	23	609	3.8%	59	7,348	0.8%	4.8(2.97–7.91)	<0.001
0.14	22	545	4.0%	60	7,412	0.8%	5.2(3.14–8.47)	<0.001
0.15	22	499	4.4%	60	7,458	0.8%	5.7(3.46–9.35)	<0.001
0.16	22	465	4.7%	60	7,492	0.8%	6.2(3.74–10.12)	<0.001
0.17	22	411	5.4%	60	7,456	0.8%	7.1(4.28–11.62)	<0.001
0.18	22	384	5.7%	60	7,573	0.8%	7.6(4.62–12.54)	<0.001
0.19	21	355	5.9%	61	7,602	0.8%	7.8(4.68–12.92)	<0.001
0.20	19	329	5.9%	63	7,628	0.8%	7.4(4.35–12.45)	<0.001

ΔSI = delta shock index; CI = confidence interval; OR = odds ratio.

## Discussion

This study revealed that the accuracy with which the ΔSI predicted a requirement for MT was not satisfactory. However, it is noted that patients had a significant higher need for MT when the patients had a ΔSI ≥ 0.00 and, as the ΔSI increased, the odds of a need for MT in patients with ΔSI ≥ a given value significantly increased. This result may also imply that an increase in HR under same value of SBP of the patients suggests a higher odd of need for MT. In this study, a higher SI at the ER compared to the SI in the field significantly increased the odds of a need for MT. In addition, the size of the delta is significantly associated with need for MT. The larger the gap between the SI at the ER and that in the field, the higher the odds of need for MT.

In this study, the ΔSI was not a significant independent risk factor for the requirement of MT and was also inaccurate in predicting the need for a MT; the AUC of ΔSI (AUC, 0.61) was even lower than that of SI (AUC, 0.760) and MSI (AUC, 0.756) [1]. In another study on hospitalized patients aged above 14 years with severe injury (ISS ≥ 16), the AUC of SI (AUC, 0.89) and MSI (AUC, 0.90) were higher in predicting massive bleeding in patients [7]. We believe this is because we only included patients with stable blood pressure in the ER in this study. The inclusion of those who had unstable blood pressure at the ER in the previous study population could have increased the accuracy of the prediction of the investigated variables, because the probability of MT in these patients with unstable blood pressure is higher and easy to predict. However, as performed in this study, to predict a need for MT in those trauma patients who had stable blood pressure at ER is more meaningful. For the same reason, predicting the need for MT in patients with severe injury is expected to be more accurate than that in patients with mild injury and stable blood pressure. In this study, the multivariate logistic regression analysis revealed that the only ISS were a significant independent risk factor for MT in trauma adult patients. However, because the ISS should be calculated according to the confirmatory injury in all anatomical regions and is always calculated much later after admission, the prediction of MT in those patients subdivided by a defined ISS seemed to be less useful in the clinical setting.

Some models have been in development for years to enable prediction of the need for MT. The Trauma Associated Severe Hemorrhage (TASH) score uses seven independent variables: sex, SBP, HR, level of hemoglobin and base deficit, focused assessment with sonography in trauma (FAST) results, and the presence or absence of complex long bone and/or pelvic fractures. With a score of 16 points or above indicating a probability of MT of more than 50%, the AUC for TASH is reported to be 0.892 [17, 18]. The Prince of Wales Hospital (PWH) score uses seven independent variables: HR, SBP, level of hemoglobin and base deficit, displaced pelvic fracture, a positive FAST or CT result, and Glasgow Coma Scale score [2, 19, 20]. The reported AUC of PWH is 0.889, with a sensitivity of 31.5% and specificity of 99.7% [2]. The Assessment of Blood Consumption (ABC) score did not use laboratory data with non-weighted variables, including HR, SBP, penetrating mechanism, and a positive FAST result. With sensitivity of 75% and specificity of 86%, the reported AUC of ABC is 0.852 [21, 22]. The Revised Assessment of Bleeding and Transfusion score was developed and the replacement of hypotension and tachycardia with a SI>1.0 and inclusion of pelvic fracture as a variable enhanced discrimination of ABC score for predicting the need for MT [23]. Although a higher accuracy may be acquired by the inclusion of more parameters; however, what parameters are available and able to increase the accuracy require further investigation and validation. For example, some experienced physicians might wonder about the need for a prediction for MT when laboratory data such as hemoglobin level and base deficit could be acquired with the trauma mechanism easily. An earlier indicator that does not involve laboratory testing may represent an attractive option.

According to the results of this study, we propose a simple concept that for the trauma patient who has a stable blood pressure at the ER, a patient’s SI at the ER is equal to or higher than that in recorded in the field (i.e., ΔSI ≥ 0.00) indicated there is significantly higher odds of a need for MT. The size of the delta is significantly associated with need for MT. However, some limitations should be acknowledged in this study. First, the retrospective study design may carry a selection bias, as it cannot be determined if the indication and judgment regarding the amount of blood transfused would have been the same among different physicians at the ER. Second, the volume and rate of other fluids infused during resuscitation by the EMS and at the ER is unknown, and this variation might be extensive. Third, prior medication history such as consumption of antihypertensive drugs, beta-blockers, and anti-anxiety drugs was unknown, and such drugs might influence SBP and HR and, therefore, the SI in the field or at the ER. Fourth, the requirement for massive transfusion in some patients was due to the surgical procedures, thus may lead to a bias in the assessment for the requirement of transfused blood for initial injury. An analysis under more acute definition, e.g. 5 U in 4 h or 4 U in 2 h, may reflect the requirement of blood transfusion for the initial injury. However, such analysis was limited by the available registered data and present different meaning in the preparation of large blood amount (10 U of blood but not less amount of blood) in cope with the critical condition of the patients. A prospective study with information of initial fluid resuscitation at the field, implementation and method of damage control, delayed time to hemorrhage control, and injury to body regions would be valuable in elucidating blood requirement of the patients at the ER. In addition, the study was limited to one trauma center, and the information obtained may limit its generalizability [24–26], and the cutoff values may also differ among countries or various trauma systems. Finally, the vital signs were based on values recorded by the experts of the EMS and the nurse at the triage desk; however, vital signs have a dynamic nature and the conclusions drawn from a single measurement may differ from those drawn from multiple measurements averaged over time.

## Conclusion

This study indicated that, in trauma patients with stable blood pressure at the ER, the accuracy of prediction of the requirement for MT by ΔSI is low. However, the size of the delta is significantly associated with need for MT and a lack of improvement in the patient’s SI at the ER compared to that in the field significantly increases the odds of a need for MT.
